# Oxidative Ring-Opening of Dimethylfuran in Zeolitic Imidazolate Frameworks through Computational Design

**DOI:** 10.1021/acs.jpcc.5c06617

**Published:** 2026-02-19

**Authors:** Thanh-Hiep Thi Le, Mohammad Reza Alizadeh Kiapi, Dhruv Menon, David Fairen-Jimenez, Manuel A. Ortuño

**Affiliations:** † Centro Singular de Investigación en Química Biolóxica e Materiais Moleculares (CIQUS), 430234 Universidade de Santiago de Compostela, 15782 Santiago de Compostela, Spain; ‡ The Adsorption & Advanced Materials Laboratory (A^2^ML), Department of Chemical Engineering & Biotechnology, 2152 University of Cambridge, Philippa Fawcett Drive, Cambridge CB3 0AS, U.K.; § Departamento de Química Física, 16718 Universidad de Alicante, 03080 Alicante, Spain

## Abstract

Furans are versatile feedstocks for producing valuable chemicals and fuels, with 2,5-dimethylfuran (DMF) particularly standing out for its potential in oxidative ring-opening reactions, yielding enediones. The zeolitic imidazolate framework ZIF-8 has shown promise as a mediator to achieve high selectivity, although a precise mechanistic understanding of how this heterogeneous system works is unclear. Here, we employ a computational protocol, combining a configurational search with force fields (GFN-FF) with a refinement at the periodic density functional theory (DFT) level, to model the oxidation of DMF with H_2_O_2_ and explicit MeOH solvent in the absence and presence of ZIF-8. In line with experimental observations, our results reveal that ZIF-8 suppresses overoxidation of the enedione *cf*. to results for the blank reaction. We further substituted the methyl group of ZIF-8 with other groups and tested the resulting materials in the above-mentioned reaction. We observe that vinyl-substituted ZIF emerges as the most selective material, while ZIF-H (SALEM-2) offers poor selectivity when compared with the blank reaction. A featurization study, correlating energy barriers and structural features, reveals how pore accessibility and linker geometry influence the selectivity. Our findings seek to deepen the understanding of molecular interactions in ZIFs and guide the rational design of MOFs for biomass conversion.

## Graphical abstract

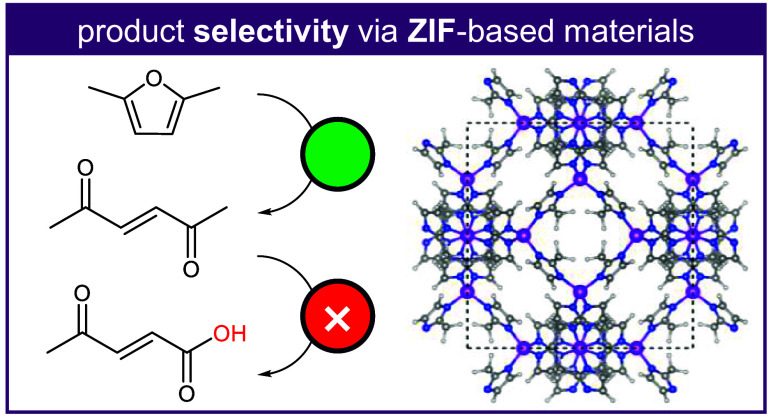

## Introduction

Biomass-derived furans are gaining significant attention as renewable feedstocks for the production of high-value chemicals and fuels.^1–4^ Their furan ring exhibits dual reactivity due to its relatively low aromaticity, enabling transformations characteristic of both aromatic compounds and alkenes.^5^ The oxidation of furans is among the most prominent methods for producing valuable aliphatic and alicyclic compounds; however, challenges persist in achieving high selectivity by minimizing undesirable byproducts, as well as in developing cost-effective and environmentally friendly oxidants and catalysts.^6–8^

The feedstock 2,5-dimethylfuran (DMF) can be easily obtained from biomass carbohydrates,^9^ and its oxidative ring-opening reaction provides direct access to enediones. The majority of early^10,11^ and recent^12,13^ reports focus on homogeneous systems, which may present limitations at industrial scales. Heterogeneous catalysts, on the other hand, are more suitable for industrial applications due to their robustness and recyclability, but research on them is scarce. Wahlen et al. reported the oxidation of DMF with the zeolite titanium silicate 1 (TS-1) and H_2_O_2_ in acetonitrile, obtaining 3-hexene-2,5-dione with 85% selectivity.^14^ Later, Miedziak et al. employed Au and Au/Pd nanoparticles and O_2_ in solvent-free conditions to convert DMF into 3-hexene-2,5-dione, obtaining up to 80% selectivity.^15^ We thus envisage room for improvement in the field of heterogeneous materials.

Inspired by the success of zeolites,^14^ we turned to metal–organic frameworks (MOFs),^16^ a family of porous materials formed by inorganic nodes connected via organic linkers. MOFs have already shown promise in biomass upgrading applications.^17,18^ In 2021, Franco et al. reported the oxidation of DMF with H_2_O_2_ in methanol to form 3-hexene-2,5-dione in the presence of the zeolitic imidazolate framework ZIF-8 with up to 85% selectivity.^19^ At a similar high DMF conversion, the blank run yielded only 27% selectivity, with 4-oxo-2-pentenoic acid as the main side product coming from a Baeyer–Villiger oxidation (Figure 1). Interestingly, performing the reaction with the unassembled ZIF-8 components (i.e., Zn^2+^ ions and 2-methylimidazolate ligands) provided only 30% selectivity, which highlights the key role of the porous network. To the best of our knowledge, this is the only MOF-based contribution to date.

**Figure 1:**
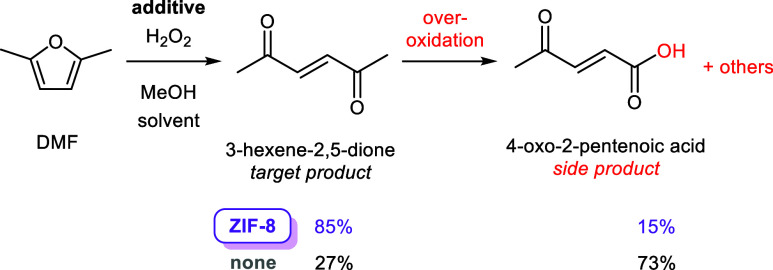
Oxidative ring-opening of DMF with H_2_O_2_ in the presence and absence of ZIF-8, including product selectivity.

These encouraging results motivated us to investigate the mechanistic details of the process involving ZIFs.^20^ These systems are amenable to linker modification,^21^ enabling precise control over pore size, framework topology, and surface chemistry, thus tuning the stability, hydrophobicity, and hydrophilicity of the materials.^22,23^ Here, we study the role of ZIF-8 in selectivity control using computational techniques.^24^ We model the oxidative ring-opening of DMF with H_2_O_2_ with explicit methanol solvent, with and without ZIF-8, as reported experimentally.^19^ We follow a multilevel approach where we first perform an exhaustive conformational search at a low level of theory (force field) and then refine the energies and structures at the quantum mechanical level (periodic density functional theory). We compute the thermodynamic profile of the reaction, considering only the key transition states that control selectivity. Finally, we investigate structural features that potentially predict the performance of other ZIFs, with the aim of guiding further experimental design.

## Computational Section

### Models

For isolated molecules, we used a cubic unit cell with a cell length of 20 Å. For ZIF-8, we employed two different approaches. We first considered a cubic unit cell with a cell length of 17.056 Å and 276 atoms, as reported in the literature (Figure 2a).^25^ From the periodic structure, we then prepared a finite-size cluster with 480 atoms to represent the full cavity, which was defined by six 4-ring and eight 6-ring windows (Figure 2b). Nodes were capped with OH^–^ and H_2_O to maintain the Zn coordination environment and charge neutrality. These groups were pointing outward of the cavity and did not interact with the host species.

**Figure 2:**
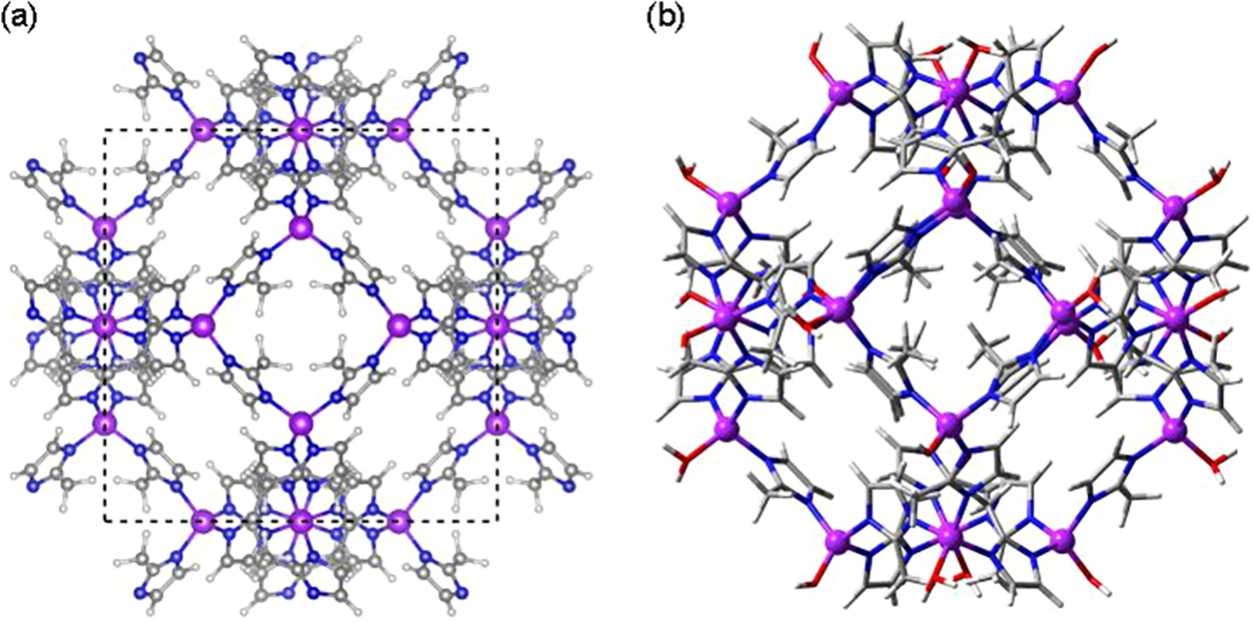
(a) Periodic structure (unit cell in dashed black lines) and (b) finite-size cluster of ZIF-8. Atom legend: Zn (purple), O (red), N (blue), C (gray), H (white).

### Methods

Our computational protocol entails a **(i)** fast but extensive conformational search with a force field using a finite-size cluster model; from there, we selected several low-energy structures and performed a **(ii)** refinement of energies and geometries at the density functional theory (DFT) level with periodic models. This strategy allows us to efficiently explore the potential energy surface and ensure that the most relevant conformers are considered. For the sake of clarity, only the most stable species are reported and discussed at the DFT level in the text. We used this protocol for both blank and MOF-mediated reactions.

Regarding the initial step **(i)**, the finite-size cluster models were computed with the GFN-FF force field^26^ as implemented in the *xtb* 6.4.1 software package.^27^ We performed an automatic conformational search with CREST^28^ for each reaction intermediate and transition state (TS) within the ZIF pores. During that search, the host framework was fixed^29^ and the guest molecules were free. In the case of TS guest molecules, selected bond distances were constrained to preserve the structure. As for the refinement step **(ii)**, the periodic models were computed in the gas phase with the PBE density functional^30^ as implemented in VASP.^31,32^ The Grimme D3 scheme^33^ with the Becke–Johnson damping function^34^ was employed to account for dispersion interactions. Core electrons were represented using projector augmented wave (PAW) pseudopotentials,^35^ and valence electrons were expanded in a plane-wave basis set with a kinetic energy cutoff of 600 eV for cell optimization. After that, the cell parameters were kept fixed and all subsequent calculations are reported with a kinetic cutoff of 450 eV. The Brillouin zone was sampled at the Γ-point, employing the Monkhorst–Pack method.^36^ TS structures were obtained using the climbing image nudged elastic band (CI-NEB)^37^ and improved dimer^38^ algorithms. Minima and TSs were characterized by diagonalizing the numerical Hessian matrix, allowing displacements of ±0.015 Å. Electronic energies were converged to 10^–6^ eV, and geometries were optimized until the forces on the atoms were less than 0.025 eV/Å. During geometry optimizations, the positions of all atoms in the system were allowed to relax without restrictions. Vibrational partition functions were computed using numerical frequencies, where only selected atoms were allowed to move. Frequencies below 100 cm^–1^ were shifted to 100 cm^–1^ when computing vibrational partition functions. All thermochemical properties were calculated using tools4VASP^39^ at 333 K, as reported experimentally.^19^

Finally, we carried out a small benchmark study of selected species for the reaction in the absence of ZIF-8. Gaussian 16 ^40^ was employed to perform single-point calculations with the def2-TZVP basis set^41^ using several density functionals with D3­(BJ) corrections (except for Minnesota functionals): PBE,^30^ BLYP,^42,43^ TPSS,^44^ M06-L,^45^ PBE0,^46^ B3LYP,^47^ TPSSh,^44,48^ and M06.^49^ ORCA 5.0 ^50^ was also employed to perform single-point calculations with the def2-TZVP basis set^41,51^ using the DLPNO–CCSD­(T)^52^ level of theory.

All energies and geometries reported herein are freely available in the open-access^53^ ioChem-BD platform^54^ through the following database.^55^

### Featurization

Atomic positions and cell parameters of ZIF candidates were optimized using periodic DFT (see above), after which their structural features were converted into fixed-sized arrays using the MOFDSCRIBE package.^56–58^ It is noted that the current model does not account for linker swing motion found in ZIF-8.^59^ Several descriptors were selected, including building unit features (inertial shape factor,^60,61^ shape of linkers,^62^ pairwise distances^56,63^ ), and pore geometry features such as accessible volume, pore diameters, and accessible surface area.^64,65^ In that respect, a radii probe of 1.7 Å (half of the diameter pore aperture in ZIF-8) was chosen. The Pearson correlation coefficient *r* was then calculated using appropriate features (see the Supporting Information for details). To visualize the correlation matrix, a Seaborn heatmap was used as implemented in the Seaborn package.^66^

## Results and Discussion

To elucidate the role of ZIF-8 as a mediator in promoting selectivity, we first computed the blank oxidation process using H_2_O_2_ in explicit MeOH solvent and compared it with that including the periodic structure of ZIF-8. We then explored the adsorption of related furan-based feedstocks. Finally, we tuned the linkers of ZIF-8 to predict the selectivity trends when using related materials as mediators. We reiterate that only the most stable structures are reported here, which were obtained after performing a configurational search at a low level (finite-size model with GFN-FF) followed by geometry and energy refinement at a high level (periodic model with PBE-D3­(BJ)).

### Blank Reaction

Figure 3 shows the main intermediates in the ring-opening oxidation of DMF to 3-hexen-2,5-dione with H_2_O_2_ in the absence of ZIF-8 (**A**). To properly describe the solvation environment, we include the solvent explicitly. After testing several models (Figure S1), we compute the reacting substrates with one methanol molecule and nonparticipating molecules with four methanol molecules. Initially, solvated DMF **A-1** (0.00 eV, energy reference) first forms an isoenergetic adduct with H_2_O_2_ **A-2** (0.06 eV). Oxidation of one double bond yields an epoxide and H_2_O **A-3** (−1.67 eV), which can further evolve via ring-opening to give 3-hexen-2,5-dione **A-4** (−2.51 eV). In the presence of excess oxidant, the reaction might continue. Removal of H_2_O in **A-5** (−2.41 eV) and addition of a second equivalent of H_2_O_2_ in **A-6** (−2.48 eV) prepare the system for Baeyer–Villiger oxidation of the ketone group. We did not study the entire reaction, which is reported elsewhere.^67,68^ Since we are interested in selectivity, we only focused on the nucleophilic attack TS to estimate the energy required to start the pathway toward side products. Thus, from **A-6** (−2.48 eV) and after a conformational reorganization of the substrates in **A-6a** (−2.33 eV),^69^ the transition state **A-TS6** (−1.81 eV) describes the nucleophilic attack of H_2_O_2_ on the ketone group with concomitant proton transfer to yield a Criegee intermediate **A-7** (−2.56 eV). This process has a Gibbs energy barrier of 0.70 eV. Subsequent steps not computed herein would eventually yield 4-oxo-2-pentenoic acid.

**Figure 3:**
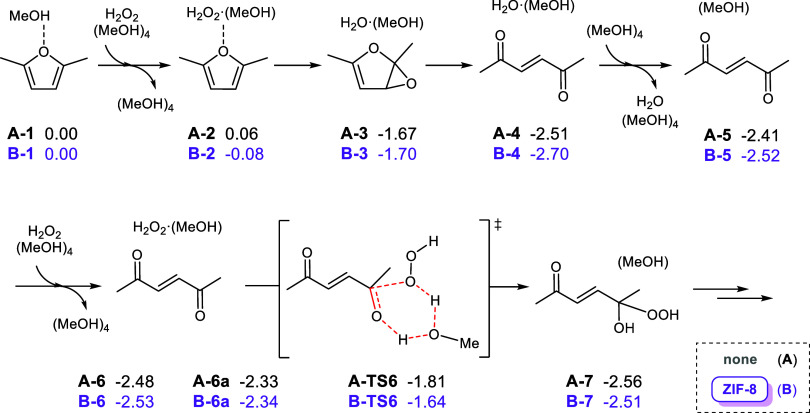
Intermediates in the ring-opening oxidation of DMF to 3-hexen-2,5-dione with H_2_O_2_ in the absence (black) and presence (purple) of ZIF-8. Gibbs energies at the PBE-D3­(BJ) level in eV.

For the blank reaction, we computed the energy barriers with several density functionals and found that GGA functionals yield lower values than hybrid ones, with those being closer to those of DLPNO–CCSD­(T) (Table S1). Since we are interested in comparing the blank reaction against the ZIF-mediated reaction, systematic errors would follow the same trend. In other words, energy barriers at PBE-D3­(BJ) may be underestimated, but the reactivity trend between systems should hold.

### ZIF-8-Mediated Reaction

After studying the blank reaction, we computed the pathway inside the pore of ZIF-8 (**B**) including the solvent explicitly. The main intermediates also shown in Figure 3 are mostly the same, except for some minor conformational changes due to the different environment. From **B-1** (0.00 eV, energy reference), the adduct **B-2** (−0.08 eV) is still isoenergetic, and the subsequent epoxide **B-3** (−1.70 eV) and dione **B-4** (−2.70 eV) follow the previous trend. Regarding the overoxidation, the thermodynamics of **B-5** (−2.52 eV), **B-6** (−2.53 eV), **B-6a** (−2.34 eV),^69^ and **B-7** (−2.51 eV) is quite similar to the blank reaction. But interestingly, the nucleophilic attack via **B-TS6** (−1.64 eV) is more energetically demanding.

For a better comparison, Figure 4 shows the Gibbs energy profiles of the blank reaction (solid black line) and the ZIF-8-mediated reaction (dashed purple line). One can see the similarity of both profiles except for two key structures: the target product 3-hexen-2,5-dione **4** and the transition state leading to overoxidation **TS6**. Inside ZIF-8, the former is lower by 0.19 eV (*cf*. blank) and the latter is higher by 0.17 eV (*cf*. blank). As a result, the net Gibbs energy barriers of overoxidation for blank and ZIF-8-mediated reactions are 0.70 and 1.06 eV, respectively; that is, the formation of side products is disfavored by 0.36 eV when the framework is present.

**Figure 4:**
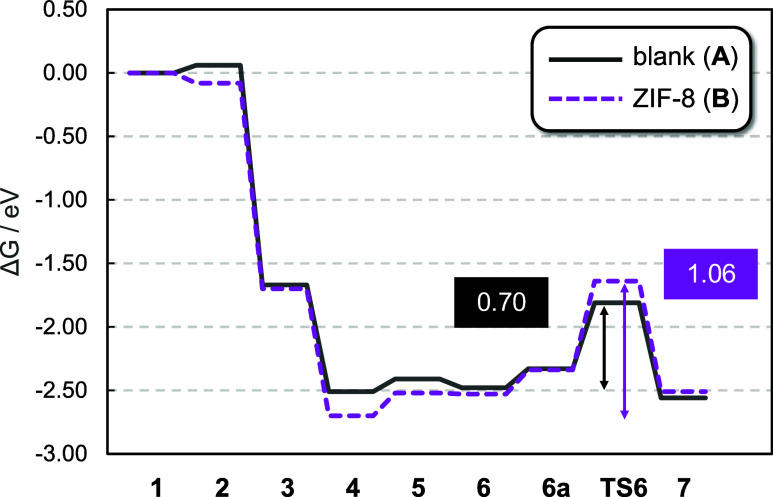
Gibbs energy profiles of the ring-opening oxidation of DMF without (black line) and with ZIF-8 (purple line). Intermediates are shown in Figure 3. Gibbs energies at the PBE-D3­(BJ) level in eV.

To further explore the origin of these differences, Figure 5 shows the optimized structures of key species **4** and **TS6** (additional structures can be consulted in Figure S2). On the one hand, intermediate **4** is slightly more stable inside ZIF-8 than in its absence by 0.19 eV. We attribute this trend to the presence of noncovalent interactions.^70^ Due to the hydrophobicity of the pore,^71^ these dispersion-driven interactions can also be extrapolated to other intermediates. On the other hand, transition state **TS6** is slightly more disfavored inside ZIF-8 than in its absence by 0.17 eV. This could be related to the need of space to adopt a rather organized transition state.^72^ Having said that, it is worth noting that the individual differences are not particularly large (less than 0.2 eV); thus, it is not straightforward to pinpoint the precise interactions that create such a trend. Nevertheless, we believe that this is not related to configurational noise, as we have previously explored the chemical space in a thorough way.

**Figure 5:**
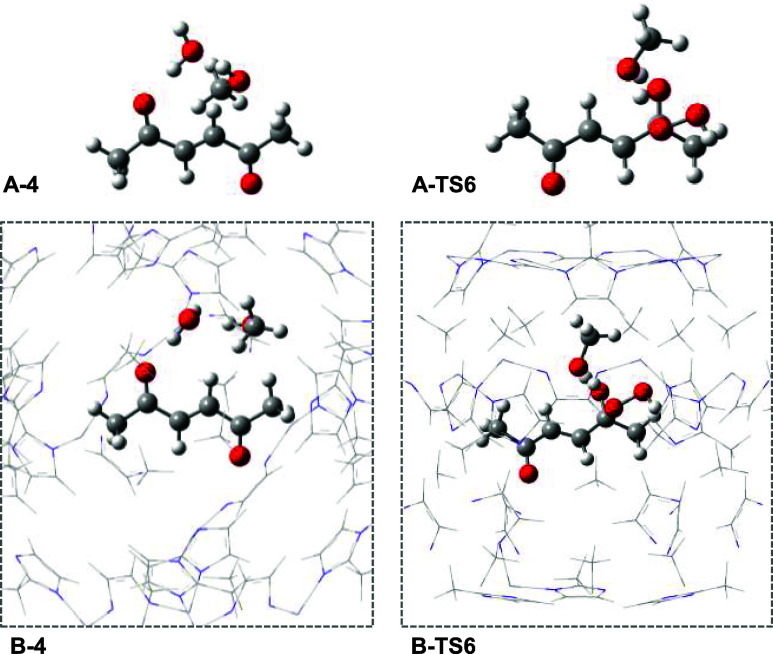
PBE-D3­(BJ)-optimized structures of intermediate 4 and **TS6** in the absence (**A**) and presence (**B**) of ZIF-8.

### Behavior of Other Furans

Although experimental work has mainly focused on the oxidation of DMF, the reactivity of other furans such as furfural (FUR), furfuryl alcohol (FAL), and 5-(hydroxymethyl)­furfural (HMF) was also tested, but with no success.^19^ Thus, we wondered whether our current model could provide a qualitative explanation for this observation. While computing the full thermodynamics for all variants is out of the scope of the present work, we did, however, consider their initial adsorption inside the ZIF-8 pores. Following the previous protocol and model, we computed the reaction energy associated with the adsorption of furans solvated by 4 MeOH molecules, as shown in Figure 6. The resulting Gibbs energy differences were −0.14, 0.08, 0.32, and 0.39 eV for DMF, FUR, FAL, and HMF, respectively. It is worth noting that only the step involving DMF is exoergic, while the other compounds are isoenergetic or endoergic. Despite the simplicity of these calculations, they already provide a reasonable trend for furans, showing that more hydrophilic species are less likely to interact with the framework, as suggested experimentally.

**Figure 6:**
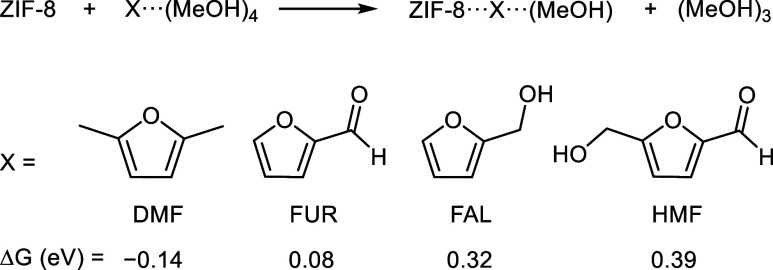
Reaction Gibbs energies for the adsorption of solvated furans inside ZIF-8. Gibbs energies at the PBE-D3­(BJ) level in eV.

### Prediction of Selectivity Enhancement

Building on the previous results that can qualitatively simulate the experimental trends, we now investigate whether other ZIFs can enhance the selectivity of the reaction. Previous studies have pointed out the impact of linker exchange on the interaction between host material and guest molecule.^73^ Thus, we prepared six ZIF-based structures (with the same sodalite topology) using 2-substituted imidazolate linkers, where −CH_3_ was exchanged by different organic groups. We employed –C≡CH,^74^ −CH=CH_2_,^75^ −Br,^76,77^ −Cl,^76,77^ −H,^78^ and −CHO,^79^ referring to them as ZIF-Ethynyl, ZIF-Vinyl, ZIF-Br, ZIF-Cl, ZIF-H (also SALEM-2), and ZIF-CHO (also ZIF-90), respectively (Figure 7a). For computational efficiency, we used the ZIF-8 unit cell for the six modified ZIFs as the linker substitutions were relatively minor.^80^ With the new candidates in hand, we computed the adsorption energies following the scheme in Figure 6. All processes were exoergic with values of −0.29, −0.21, −0.36, −0.19, −0.16, and −0.30 eV, respectively. We then computed the simplified Gibbs energy profiles (Figure S3) and the Gibbs energy barriers for the overoxidation (Figure 7b). Taken as reference the blank value of 0.70 eV, all materials perform better except for ZIF-H with 0.75 eV, from which low selectivity would be expected. Taken as reference the ZIF-8 value of 1.06 eV, most materials perform slightly worse except for ZIF-CHO with 1.11 eV and ZIF-Vinyl with 1.27 eV, for which similar and enhanced selectivities would be expected. These barriers seem to be influenced by more than just the electron-donating or -withdrawing nature of the substituents, pointing to other properties that might also play a role. Thus, we next study the impact of linker modifications on both the topology of linkers and the pore structure in further detail.

**Figure 7:**
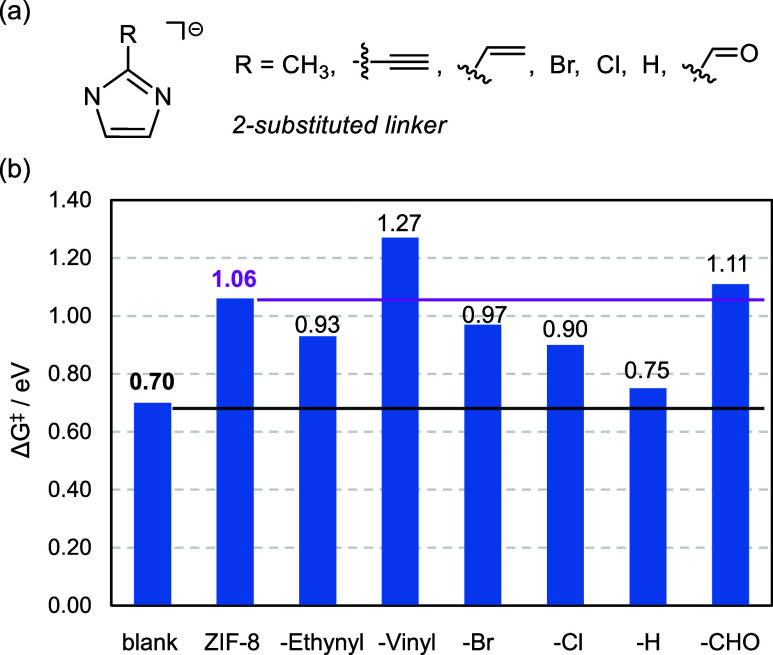
(a) Linker modifications of ZIF materials and (b) Gibbs energy barriers of overoxidation for the blank and all of the materials. Gibbs energies at PBE-D3­(BJ) level in eV.

### Correlating Energy Barriers and Structural Properties

In an attempt to rationalize structure–property relationships that affect the energy barriers, we performed a featurization study of the designed MOFs to extract the main descriptors using MOFDSCRIBE.^56^ Different features were selected to describe the pore properties, including probe accessible volume, pore diameters, and probe accessible surface areas, along with the chemistry of the building unit, including inertial shape factor and shape of linkers (only linkers were displayed due to unchanged Zn node). Previous computed adsorption energies are also included. A detailed explanation of all descriptors can be found elsewhere.^57,58^

All data are collected as a heatmap in Figure 8. We first note that there is a negligible correlation between the energy barriers and the adsorption energies. We then observed that, when using a 1.7 Å probe, accessible volume values of the ZIFs are divided into two groups: one that allows access (ZIF-8, ZIF-Vinyl, and ZIF-CHO), and another one that does not (ZIF-Ethynyl, ZIF-Br, ZIF-Cl, and ZIF-H). The accessible group exhibits a strong positive correlation with energy barriers, and the same trend is also observed for the accessible surface area. For pore diameters, we calculated the largest included sphere (lis), largest free sphere (lifs), and largest included sphere along free sphere path (lifsp).^81^ ZIF-Vinyl has the highest lifs value (3.67 Å), followed by ZIF-CHO (3.49 Å) and ZIF-8 (3.43 Å), and the least lifs value is found in ZIF-H (3.18 Å), resulting in differences in how freely guest molecules can access the pores. Then, we explored the correlation between linker shape features; specifically, rod- and sphere-like features are positively correlated with energy barriers, whereas disk-likeness shows a negative correlation. Additionally, we computed a histogram and statistical analysis of pairwise distances (Figure S4). The mean pairwise interatomic distances are 8.29 Å for ZIF-Vinyl, 8.24 Å for ZIF-CHO, 8.20 Å for ZIF-8, and 8.14 Å for ZIF-Ethynyl, with the smallest value of 8.08 Å found in ZIF-H.

**Figure 8:**
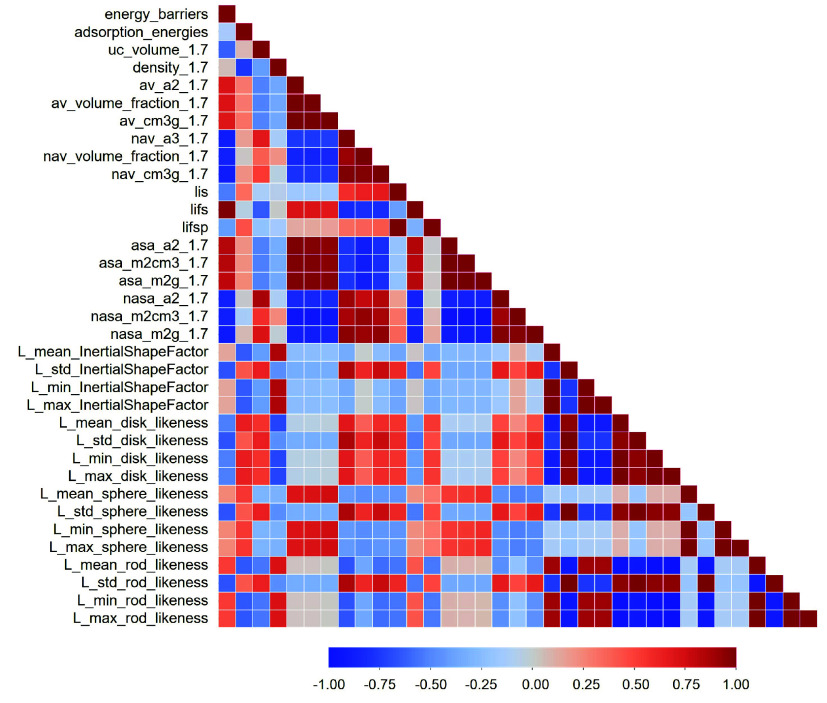
Correlation diagram from the featurization of seven ZIF-based materials, including Gibbs energy barriers. L = linker.

These results highlight how linker substitutions can alter the size, shape, and distribution of pore apertures, which subsequently impact guest molecule diffusion and adsorption. This, in turn, influences the energy barriers, ultimately helping to reduce the formation of side products and improve selectivity control. Nevertheless, we point out that our modest MOF sample size could limit such correlations, and additional data would be required to make more robust predictions.

## Conclusions

Our study investigates the ring-opening oxidation of DMF mediated by various ZIF-based materials. Simulations show that, while the oxidation is favorable both in the presence or absence of ZIF-8, the overoxidation is reduced within the framework, in agreement with available experimental data. Among the new materials tested for this reaction, vinyl-substituted ZIF exhibits the best performance, and ZIF-H exhibits the worst one. A subsequent featurization study indicates that the accessible volume and surface area are strongly correlated with energy barriers, where ZIF-H exhibits the highest nonaccessible volume and surface area, in line with the predicted suboptimal performance. We also observe that linkers with a more linear or elongated configuration, resembling between rod-likeness and sphere-likeness, contribute to enhance selectivity. These insights provide valuable knowledge for future experimental testing and the rational design of MOFs for the conversion of furans, paving the way for improved efficiency and selectivity.

## Supplementary Material



## Notes

Additional reaction profiles and statistical analysis (PDF)
