# Supplementary material for: Oxidative Ring-Opening of Dimethylfuran in Zeolitic Imidazolate Frameworks through Computational Design
Source: J Phys Chem C Nanomater Interfaces. 2026 Feb 19;130(9):3245–52. doi: 10.1021/acs.jpcc.5c06617 (PMC12969367; doi:10.1021/acs.jpcc.5c06617)
Supplement: Supplementary file 1 [file jp5c06617_si_001.pdf]

SUPPORTING INFORMATION

Oxidative Ring-Opening of Dimethylfuran in Zeolitic Imidazolate Frameworks through Computational Design

Thanh-Hiep Thi Le,[a] Mohammad Reza Alizadeh Kiapi,[b] Dhruv Menon,[b] David Fairen-Jimenez,[b] Manuel A. Ortuño*,[a,c]

[a] Centro Singular de Investigación en Química Biolóxica e Materiais Moleculares (CIQUS), Universidade de Santiago de Compostela, 15782 Santiago de Compostela, Spain

[b] The Adsorption & Advanced Materials Laboratory (A^2ML), Department of Chemical Engineering & Biotechnology, University of Cambridge, Philippa Fawcett Drive, Cambridge CB3 0AS, U.K.

[c] Departamento de Química Física, Universidad de Alicante, 03080 Alicante, Spain

***Corresponding Authors:**

Dr. Manuel A. Ortuño, E-mail: manuel.ortuno@ua.es

Contents

1. DMF oxidation in blank reaction with explicit $(MeOH)_n$ solvent S3
2. Additional figures for DMF oxidation in the presence of ZIF-8 .... S4
3. Evaluation of density functionals S5
4. Simplified Gibbs energy profiles in six modified ZIFs S6
5. Correlation matrix S6
6. Histogram and statistics of pairwise distances S7
7. References S8

1. DMF oxidation in blank reaction with explicit $(MeOH)_n$ solvent

The ring-opening oxidation of DMF using H_2O_2 in MeOH solvent without ZIF-8 mediator is investigated, and its proposed reaction pathway is illustrated in Figure S1a. The reacting substrate is modeled with one MeOH molecule, while non-participating molecules are explicitly solvated with $(MeOH)_n$, where n represents the number of solvation molecules ranging from 1 to 4. Figure S1b presents the Gibbs energy profiles corresponding to this mechanism. Notably, from **A-6** to **A-7**, we observe that the relative Gibbs energies converge with $(MeOH)_4$, making this model the preferred choice for further investigation.

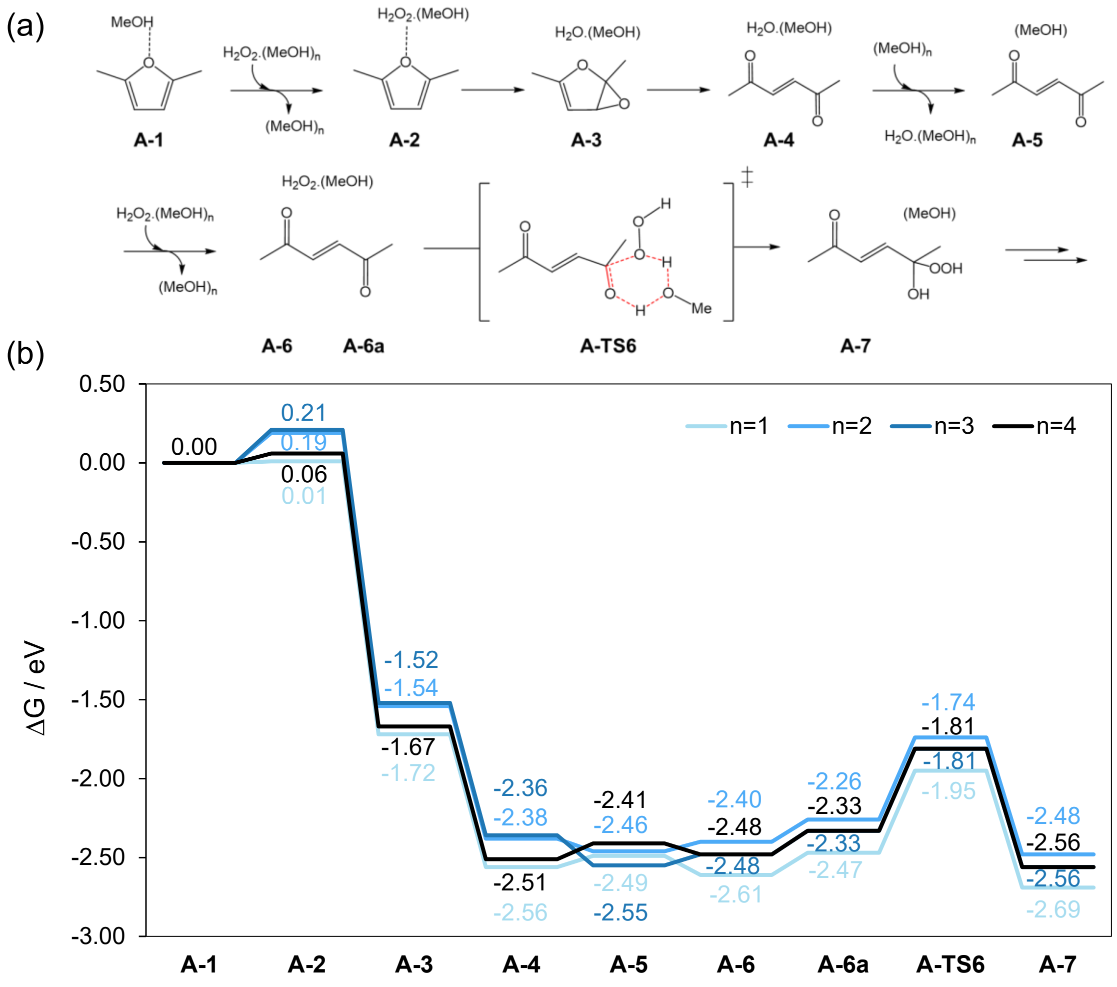

Figure S1. (a) Intermediates in ring-opening oxidation of DMF to 3-hexen-2,5-dione using H_2O_2 with explicit $(MeOH)_n$ solvent; (b) Gibbs energies at PBE-D3(BJ) level in eV.

2. Additional figures for DMF oxidation in the presence of ZIF-8

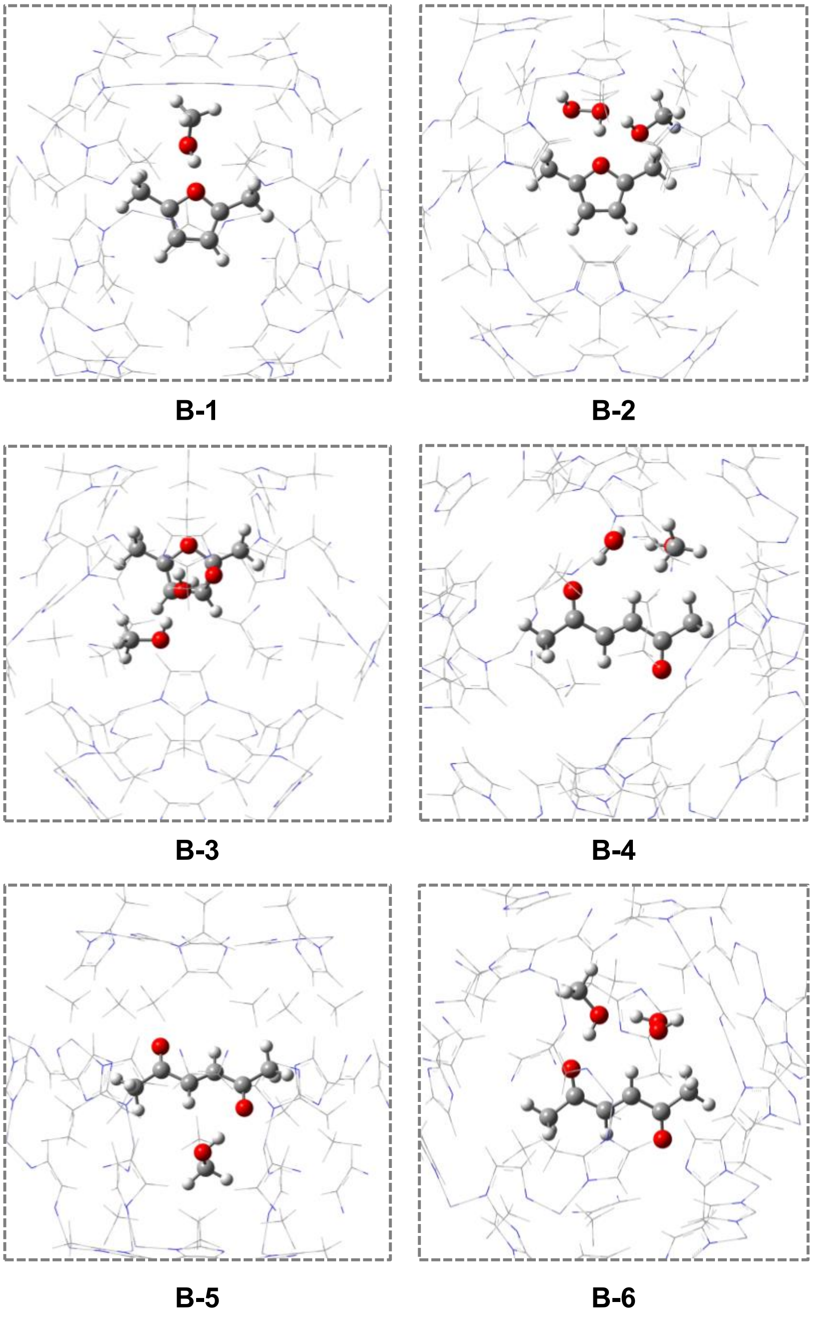

Figure S2. PBE-D3(BJ)-optimized structures of intermediates in the presence of ZIF-8.

3. Evaluation of density functionals

Plane-wave-based DFT calculations usually limit the variety of density functionals that can be used due to high computational costs. Thus, in order to assess the impact of the density functional, we perform single-point calculations on molecular models of the blank reaction, i.e., without ZIF-8. We employed the def2-TZVP basis set and several density functionals with D3(BJ) corrections as implemented in Gaussian 16. We tested two GGA (PBE, BLYP), two meta-GGA (TPSS, M06-L) and four hybrid (PBE0, B3LYP, TPSSh, M06) density functionals. We also run DLPNO-CCSD(T) single-point with def2-TZVP as implemented in ORCA 5.0. The resulting electronic energy barriers (from **A-4** to **A-TS6**) are shown in Table S1.

Table S1. Electronic energy barriers at different levels of theory in eV.

**Method**	PBE[a]	PBE	BLYP	TPSS	M06-L
**$\Delta E^{\ddagger}$ / eV**	0.75	0.75	0.91	0.84	1.12
**Method**	PBE0	B3LYP	TPSSh	M06	DLPNO-CCSD(T)
**$\Delta E^{\ddagger}$ / eV**	1.04	1.12	0.94	1.17	1.26

[a] Periodic DFT in VASP.

In general terms, barriers with GGA functionals tend to be lower than those with hybrid functionals, while the meta-GGA M06-L exhibits a behavior similar to the latter ones. However, a direct comparison with the real system is not trivial as we encounter several caveats. Firstly, there is not enough experimental data to assess which density functional performs best, although DLPNO-CCSD(T) calculations suggest that hybrid functionals are more accurate. Secondly, the comparison between atom-centered and plane-wave basis sets with different software packages is not straightforward, especially when computing thermochemistry. Since we are interested in trends rather than absolute barriers, we believe that systematic errors would follow the same trend when comparing barriers between the blank reaction and the ZIF-mediated reaction.

4. Simplified Gibbs energy profiles in six modified ZIFs

Figure S3 presents the simplified Gibbs energy profiles computed for species **C-1**, **C-2**, **C-4**, and **C-TS6** inside the pores of six ZIF-based structures, where C represents ZIF-Ethynyl, ZIF-Vinyl, ZIF-Br, ZIF-Cl, ZIF-H, and ZIF-CHO.

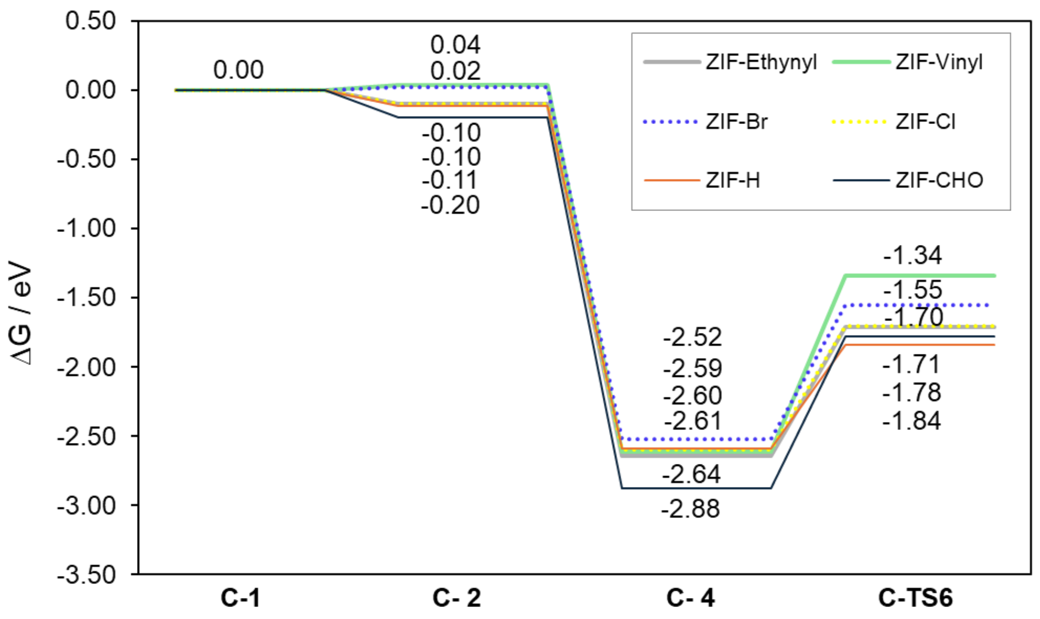

Figure S3. Simplified Gibbs energy profiles for selected steps in DMF oxidation using six ZIF mediators. Gibbs energies at PBE-D3(BJ) level in eV.

5. Correlation matrix

The Pearson correlation coefficient *r* was then calculated using appropriate features, which varies between -1 (indicating a negative correlation) and +1 (indicating a positive correlation), as described below:

$$r = \frac{\sum_{i=1}^{n}(x_i - \bar{x})(y_i - \bar{y})}{\sqrt{\sum_{i=1}^{n}(x_i - \bar{x})^2}\sqrt{\sum_{i=1}^{n}(y_i - \bar{y})^2}}$$

where *i* represents the index for each descriptor in the dataset; x_i, y_i are the values of the two different features being compared; $\bar{x}, \bar{y}$ are their respective mean values.

6. Histogram and statistics of pairwise distances

The computed descriptors include histograms and statistics of pairwise distances (*i.e.*, collection of distances between all atoms in a structure) for all studied ZIFs materials as implemented in MOFDSCRIBE.[1,2] Figure S4 top shows the mean distance and standard deviation (STD); Figure S4 bottom shows the number of items (distances) occurring in intervals of 0.5 Å (from 1.0 Å to 14.5 Å).

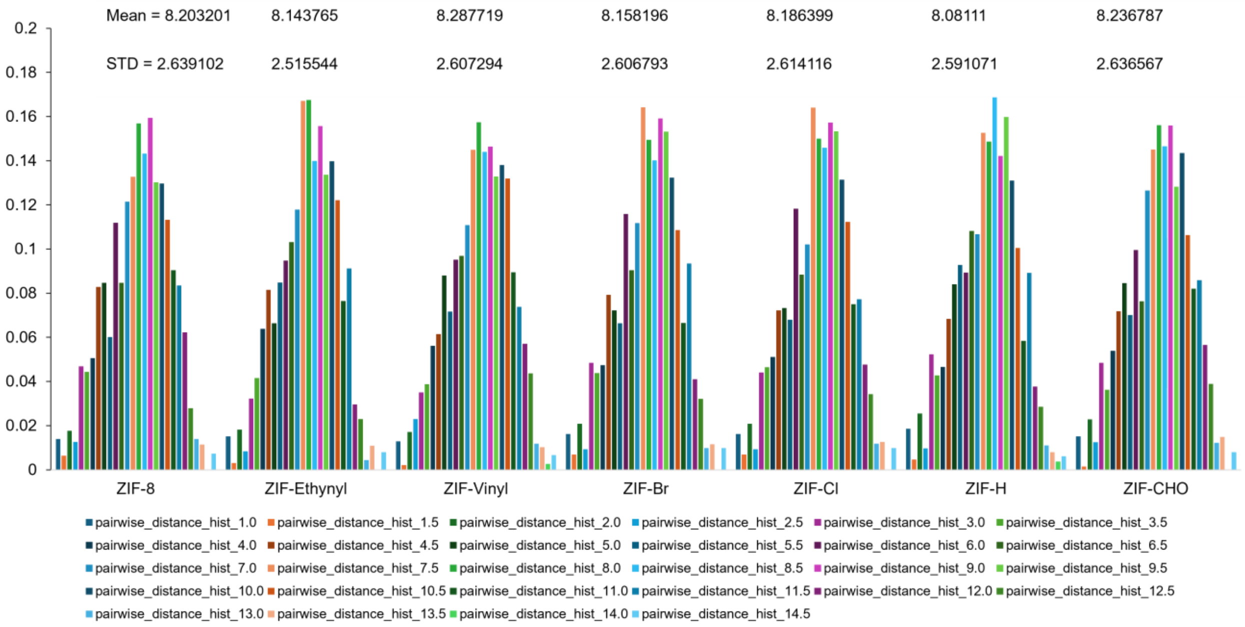

Figure S4. Pairwise distances analysis with statistics and histograms for all ZIF structures. Distances in Å.

7. References

1. Jablonka, K. M.; Rosen, A. S.; Krishnapriyan, A. S.; Smit, B. An Ecosystem for Digital Reticular Chemistry. *ACS Cent. Sci.* **2023**, *9*, 563-581, DOI: 10.1021/acscentsci.2c01177

2. Zhang, R. Z.; Seth, S.; Cumby, J. Grouped Representation of Interatomic Distances as a Similarity Measure for Crystal Structures. *Dig. Discov.* **2023**, *2*, 81-90, DOI: 10.1039/D2DD00054G
